# Effects of Amount, Intensity, and Mode of Exercise Training on Insulin Resistance and Type 2 Diabetes Risk in the STRRIDE Randomized Trials

**DOI:** 10.3389/fphys.2021.626142

**Published:** 2021-02-04

**Authors:** Leanna M. Ross, Cris A. Slentz, Alyssa M. Zidek, Kim M. Huffman, Irina Shalaurova, James D. Otvos, Margery A. Connelly, Virginia B. Kraus, Connie W. Bales, Joseph A. Houmard, William E. Kraus

**Affiliations:** ^1^Department of Medicine, Duke Molecular Physiology Institute, Duke University School of Medicine, Durham, NC, United States; ^2^Laboratory Corporation of America Holdings (LabCorp), Morrisville, NC, United States; ^3^Center for the Study of Aging, Department of Medicine, Duke University School of Medicine, Durham, NC, United States; ^4^Geriatric Research, Education, and Clinical Center, Durham VA Medical Center, Durham, NC, United States; ^5^Department of Kinesiology, East Carolina University, Greenville, NC, United States

**Keywords:** biomarkers, cardiometabolic health, glucose homeostasis, lifestyle intervention, lipoproteins, nuclear magnetic resonance spectroscopy, physical activity

## Abstract

**Background:**

Lipoprotein Insulin Resistance Index (LP-IR) and Diabetes Risk Index are novel spectroscopic multimarkers of insulin resistance and type 2 diabetes risk. As the Studies of a Targeted Risk Reduction Intervention through Defined Exercise (STRRIDE) randomized trials have previously demonstrated the ability of exercise training to improve traditional markers of insulin action, the aim of this study was to examine the effects of exercise amount, intensity, and mode on LP-IR and the Diabetes Risk Index.

**Methods:**

A total of 503 adults with dyslipidemia [STRRIDE I (*n* = 194), STRRIDE AT/RT (*n* = 139)] or prediabetes [STRRIDE-PD (*n* = 170)] were randomized to control or one of 10 exercise interventions, ranging from doses of 8–23 kcal/kg/week; intensities of 50–75% V̇O_2peak_; and durations of 6–8 months. Two groups included resistance training and one included dietary intervention (7% weight loss goal). Fasting plasma samples were obtained at baseline and 16–24 h after the final exercise bout. LP-IR, the Diabetes Risk Index, and concentrations of the branched chain amino acids valine and leucine were determined using nuclear magnetic resonance spectroscopy. LP-IR and the Diabetes Risk Index scores range from 0–100 and 1–100, respectively (greater scores indicate greater risk). Paired t-tests determined significance within groups (*p* < 0.05).

**Results:**

After training, six exercise groups significantly improved LP-IR (ranging from −4.4 ± 8.2 to −12.4 ± 14.1), and four exercise groups significantly improved the Diabetes Risk Index (ranging from −2.8 ± 8.2 to −8.3 ± 10.4). The most beneficial interventions for both LP-IR and the Diabetes Risk Index were low amount/moderate intensity aerobic, aerobic plus resistance, and aerobic plus diet.

**Summary:**

Multiple exercise interventions improved LP-IR and the Diabetes Risk Index. In those with dyslipidemia, adding resistance to aerobic training elicited a synergistic effect on insulin resistance and type 2 diabetes risk. In individuals with prediabetes, combining a dietary intervention and weight loss with aerobic training resulted in the most robust type 2 diabetes risk improvement.

## Introduction

Insulin resistance is one of the core pathophysiologic contributors to the progression from normoglycemia and/or prediabetes to type 2 diabetes (Defronzo, 2009). The hallmarks of insulin resistance include increased fasting insulin and changes in the lipid profile, such as increased triglycerides and decreased high density lipoprotein (HDL) cholesterol. Insulin resistance is also characterized by specific alterations in lipoprotein subclass and size parameters including increased large very low density lipoprotein (VLDL), small low density lipoprotein (LDL), and VLDL size, and decreased large HDL, LDL size, and HDL size (Frazier-Wood et al., 2012; Shalaurova et al., 2014). Furthermore, insulin resistance and other obesity-associated conditions – such as type 2 diabetes and cardiovascular disease – are often accompanied by dysmetabolism, which can manifest as increased branched chain amino acid concentrations (Newgard et al., 2009; Newgard, 2012; Lynch and Adams, 2014; Yoon, 2016; Holecek, 2018). Alterations in branched chain amino acids and the lipoprotein profile tend to become exacerbated during the progression to frank type 2 diabetes. Thus, assessing these biomarkers clinically not only helps stratify risk for type 2 diabetes, but also monitor disease progression and response to preventive treatment.

The Lipoprotein Insulin Resistance Index (LP-IR) is a nuclear magnetic resonance spectroscopy-based multimarker score calculated from six lipoprotein subclass and size parameters altered by insulin resistance (Shalaurova et al., 2014). LP-IR is strongly associated with common measures of insulin resistance, such as homeostatic model assessment of insulin resistance (HOMA-IR) and glucose disposal rate calculated from a hyperinsulinemic-euglycemic clamp (Shalaurova et al., 2014), and several large observational studies found LP-IR predicts incident type 2 diabetes (Mackey et al., 2015; Dugani et al., 2016; Harada et al., 2017; Flores-Guerrero et al., 2019). Combining LP-IR with the branched chain amino acids valine and leucine yields the recently developed Diabetes Risk Index, which has recently been found to enhance type 2 diabetes risk stratification even among individuals with glucose levels in the normoglycemic or prediabetic ranges (Flores-Guerrero et al., 2020). As insulin resistance and dysmetabolism are both related to the progression to type 2 diabetes (Flores-Guerrero et al., 2018, 2019), the Diabetes Risk Index provides a simple clinical score to improve disease risk assessment.

The three Studies of a Targeted Risk Reduction Intervention through Defined Exercise (STRRIDE) randomized trials provide a unique opportunity to assess the effects of different amounts, intensities, and modes of exercise on LP-IR and the Diabetes Risk Index. STRRIDE I (NCT00200993) investigated the separate effects of amount and intensity of aerobic exercise on cardiometabolic risk factors in adults with dyslipidemia. Subsequently, STRRIDE AT/RT (NCT00275145) examined the independent and combined effects of aerobic and resistance exercise on cardiometabolic health. Finally, STRRIDE-PD (NCT00962962) evaluated the effects of different amounts and intensities of aerobic exercise – with and without weight loss – in adults with prediabetes. Notably, STRRIDE-PD included a group similar to the lifestyle arm of the Diabetes Prevention Program, which was a multicenter randomized clinical trial designed to evaluate if weight loss through exercise plus dietary changes would prevent or delay the onset of diabetes in high risk individuals (Knowler et al., 2002). Mimicking current clinical guidelines for prediabetes, this group allowed us to determine how much of this “gold standard” effect was achieved with exercise alone. Overall, several STRRIDE interventions improved traditional markers of insulin action, and the magnitudes of these improvements were related to the specific amount, intensity, and mode of exercise performed (Houmard et al., 2004; AbouAssi et al., 2015; Slentz et al., 2016). Some of the main insulin action findings resulted from intravenous and oral glucose tolerance tests (OGTTs), which are costly and require significant participant commitment. Since LP-IR and the Diabetes Risk Index are conveniently derived from a single fasted blood sample, exploring whether these markers are beneficially altered following structured exercise intervention is clinically important. Therefore, we sought to determine whether our previously documented effects of exercise amount, intensity, and mode extend to these new spectroscopically-derived markers of insulin resistance and type 2 diabetes risk.

## Materials and Methods

### STRRIDE Study Participants

Changes in markers of insulin action, dysmetabolism, and diabetes risk were assessed in participants from STRRIDE I (Kraus et al., 2001), STRRIDE AT/RT (Slentz et al., 2011), and STRRIDE-PD (Slentz et al., 2016). STRRIDE I (1999–2003) and STRRIDE AT/RT (2004–2008) enrolled previously sedentary, overweight or obese men and women with mild-to-moderate dyslipidemia (classified by LDL-cholesterol: 130–190 mg/dL or HDL-cholesterol: ≤40 mg/dL for men and ≤45 mg/dL for women). STRRIDE-PD (2009–2012) enrolled previously sedentary, overweight or obese men and women with pre-diabetes (defined by two consecutive fasting glucose concentrations ≥95 to <126 mg/dL taken 1 week apart).

In STRRIDE I, participants were randomized into one of four groups for 8 months: (1) inactive control; (2) low amount/moderate intensity aerobic exercise: 14 kcal/kg of body weight/week (KKW) at 40–55% peak oxygen consumption (V̇O_2peak_)_;_ (3) low amount/vigorous intensity aerobic exercise: 14 KKW at 65–80% V̇O_2peak_; and (4) high amount/vigorous intensity aerobic exercise: 23 KKW at 65–80% V̇O_2peak_ (Kraus et al., 2001). In STRRIDE AT/RT, participants were randomized to one of three groups for 8 months: (1) aerobic training only: 14 KKW at 65–80% V̇O_2peak_; (2) resistance training only: 3 days/week, eight exercises, three sets/exercise, 8–12 repetitions/set; and (3) full combination of the aerobic and resistance training programs (Slentz et al., 2011). Finally, in STRRIDE-PD, participants were randomized to one of four aerobic training groups for 6 months: (1) low amount/moderate intensity: 11 KKW at 40–55% V̇O_2reserve_; (2) high amount/moderate intensity: 17.5 KKW at 40–55% V̇O_2researve_; (3) high amount/vigorous intensity: 17.5 KKW at 65–80% V̇O_2reserve_; and (4) clinical lifestyle, which included low amount/moderate intensity exercise of 11 KKW at 40–55% V̇O_2reserve_ plus diet to achieve 7% body weight loss (Slentz et al., 2016).

Both STRRIDE I and AT/RT study protocols were approved by the institutional review boards at Duke University and East Carolina University. The STRRIDE-PD study protocol was approved by the institutional review board at Duke University. Participants provided both verbal and signed written informed consent.

### Laboratory Measurements

Participants underwent multiple laboratory measures at both baseline and post-intervention. Body mass and height were assessed while participants were wearing light clothing and no shoes.

Under medical supervision, participants completed graded maximal cardiopulmonary exercise tests on a treadmill with expired gas analysis (TrueMax Parvomedics; Provo, UT, United States) and 12-lead electrocardiography. The graded treadmill test protocol consisted of 2-min stages, starting at 3 mph and 0% grade, and then increased speed and/or grade by approximately one metabolic equivalent per stage until the participant reached volitional exhaustion (Duscha et al., 2005). The same exercise testing protocol was used for all three STRRIDE trials. To determine V̇O_2peak_, the two greatest, consecutive 15-s readings were averaged.

Fasting blood samples were collected at baseline and 16–24 h after the final exercise bout. For STRRIDE I and STRRIDE AT/RT, fasting blood samples were obtained from the beginning of a 3-h intravenous glucose tolerance test (IVGTT). Fasting plasma glucose was determined *via* a YSI 2300 analyzer (Yellow Springs, OH, United States) and plasma insulin was determined *via* immunoassay (Access Immunoassay System, Beckman Coulter, Fullerton, CA, United States). HOMA-IR was calculated as {[fasting insulin (uU/mL) × fasting glucose (mg/dL)]/405}. Insulin sensitivity index was determined from the IVGTT.

During STRRIDE-PD, fasting blood samples were obtained from the beginning of a 2-h OGTT. Plasma glucose concentrations were determined with a Beckman–Coulter DxC600 clinical analyzer (Brea, CA, United States), plasma insulin was measured by electrochemiluminescent plate assay (Meso Scale Discovery, Gaithersburg, MD, United States), and HOMA-IR was calculated as described above. Matsuda index was calculated from the OGTT as previously described (Matsuda and DeFronzo, 1999).

For all three STRRIDE studies, fasted plasma samples were analyzed on 400 MHz nuclear magnetic resonance Profilers at LipoScience, now LabCorp (Morrisville, NC, United States), as previously described (Jeyarajah et al., 2006). The lipoprotein parameters as well as the branched chain amino acids were calculated by retrospectively analyzing digitally stored spectra using the newly developed LP4 algorithm (Wolak-Dinsmore et al., 2018; Berends et al., 2019; Kinzer et al., 2019; Makri et al., 2019). As previously described (Shalaurova et al., 2014), LP-IR is a composite index calculated from the results of the following six lipoprotein parameters: large VLDL, small LDL, and large HDL subclass concentrations and VLDL, LDL, and HDL size. LP-IR scores range from 0 (most insulin sensitive) to 100 (most insulin resistant). The Diabetes Risk Index is a multimarker index composed of LP-IR, valine, and leucine. As described previously (Flores-Guerrero et al., 2020), the Diabetes Risk Index was developed using logistic regression and prospective type 2 diabetes data from Multi-Ethnic Study of Atherosclerosis (MESA) (Mackey et al., 2015). Diabetes Risk Index scores range from 1 to 100, the latter representing those at greatest risk for type 2 diabetes.

### Intervention Details

For the aerobic training groups, participants underwent an initial ramp period of 2–3 months to allow gradual adaptation to their exercise prescription. The ramp period was followed by four to six additional months of training at the appropriate exercise prescription. Prescribed exercise intensity was based on each participant’s baseline cardiopulmonary exercise test results. Aerobic exercise modes included primarily treadmills, as well as elliptical trainers, cycle ergometers, or any combination of these.

The ramp period for the resistance training groups started with one set during weeks 1–2, two sets during weeks 3–4, and built up to the three-set prescription on week 5. The prescription included three sessions per week (non-consecutive days) of three sets of 8–12 repetitions on eight Cybex weight-lifting machines, designed to target all major muscle groups. To ensure a progressive stimulus throughout the intervention, the amount of weight lifted was increased by 2.75 kg each time the participant properly performed 12 repetitions on all three sets during two consecutive workout sessions.

Exercise intensity and duration for all aerobic exercise sessions were verified by direct supervision and/or with the use of downloadable heart rate monitors (Polar Electro, Woodbury, NY, United States). Aerobic training adherence was calculated for each participant as the number of minutes completed within the prescribed heart rate range, divided by the number of total weekly minutes prescribed. Resistance training sessions were verified by direct supervision and/or the FitLinxx Strength Training Partner (FitLinxx, Norwalk, CT, United States). The “training partner” automatically sent data from each session to the FitLinxx server computer. The computers recorded total weight lifted *via* laser weight plate detection and total number of repetitions (which were only counted when participants lifted through the full range of motion), and finally, speed of weight lifting motion was monitored and alerts were given when participants lifted too quickly.

Participants in the clinical lifestyle group in STRRIDE-PD received an intervention modeled after the Diabetes Prevention Program (Knowler et al., 2002). This group was designed to achieve 7% weight loss *via* energy-intake restriction, low-fat diet, and exercise. These participants attended four initial counseling sessions, followed by 12 bi-weekly intensive group sessions adapted from the Diabetes Prevention Program manual.

### Statistical Analyses

Participants who completed the intervention and had pre- and post-intervention fasting plasma samples available for analysis were included (*n* = 503). Baseline characteristics were summarized as either means and standard deviations (continuous variables) or frequencies (categorical variables). Paired t-tests determined post- minus pre-intervention change score significance within groups. Cohort-specific analysis of covariance (ANCOVA), with baseline values used as covariates, determined differences among groups. A *p*-value of 0.05 was used to indicate statistical significance. Analyses were performed using SAS version 9.4 (SAS Institute, Cary, NC, United States).

## Results

Baseline characteristics for each of the three STRRIDE cohorts are presented in Table 1. Overall, participants from STRRIDE I (*n* = 194; 47.9% female; 79.9% Caucasian) were 52.4 ± 6.2 years old and had a body mass index (BMI) of 29.7 ± 3.0 kg/m^2^, an average total branched chain amino acid concentration of 396.3 ± 98.6 μmol/L, and mean LP-IR and Diabetes Risk Index scores of 54.8 ± 25.0 and 42.6 ± 20.0, respectively. Participants from STRRIDE AT/RT (*n* = 139; 56.1% female; 84.8% Caucasian) were 48.9 ± 10.3 years old and had a BMI of 30.5 ± 3.3 kg/m^2^, an average total branched chain amino acid concentration of 405.2 ± 82.0 μmol/L, and mean LP-IR and Diabetes Risk Index scores of 52.2 ± 23.6 and 44.1 ± 18.0, respectively at baseline. Overall, STRRIDE-PD participants (*n* = 170; 60.6% female; 78.2% Caucasian) were 59.3 ± 7.5 years old and had a BMI of 30.3 ± 2.8 kg/m^2^. In addition, STRRIDE-PD participants had a baseline average total branched chain amino acid concentration of 383.9 ± 72.9 μmol/L, and mean LP-IR and Diabetes Risk Index scores of 55.5 ± 21.4 and 38.7 ± 17.1, respectively. Within each STRRIDE cohort, there were no significant differences among intervention groups at baseline for the aforementioned variables.

**TABLE 1:** Baseline characteristics for STRRIDE I, STRRIDE AT/RT, and STRRIDE-PD participants.

	STRRIDE I	STRRIDE AT/RT	STRRIDE-PD
Sample size, n	194	139	170
Female,%	47.9	56.1	60.6
White,%	79.9	84.8	78.2
Age, years	52.4 (6.2)	48.9 (10.3)	59.3 (7.5)
BMI, kg/m^2^	29.7 (3.0)	30.5 (3.3)	30.3 (2.8)
Peak V̇O_2_, mL/kg/min	28.1 (5.9)	27.3 (6.0)	24.5 (5.1)
Total cholesterol, mg/dL	210.5 (37.6)	191.8 (32.8)	174.0 (27.3)
LDL cholesterol, mg/dL	129.0 (29.8)	112.7 (24.7)	98.9 (20.5)
HDL cholesterol, mg/dL	48.4 (13.1)	48.3 (12.3)	46.9 (12.2)
Triglycerides, mg/dL	158.6 (98.1)	133.9 (69.5)	128.8 (70.8)
Fasting glucose, mg/dL	93.0 (10.3)	96.4 (11.9)	105.6 (9.7)
Insulin sensitivity index, mU/L/min	3.6 (2.4)	5.1 (4.7)	-
Matsuda index	-	-	5.2 (3.2)
HOMA-IR	2.1 (1.5)	2.1 (1.2)	2.0 (1.4)
LP-IR	54.8 (25.0)	52.2 (23.6)	55.5 (21.4)
Diabetes Risk Index	42.6 (20.0)	44.1 (18.0)	38.7 (17.1)
Total BCAA, μmol/L	396.3 (98.6)	405.2 (82.0)	383.9 (72.9)
Valine, μmol/L	210.0 (49.2)	227.8 (36.3)	220.2 (37.8)
Leucine, μmol/L	147.1 (43.7)	121.1 (33.9)	117.9 (26.3)

*Values listed as means (standard deviation). BCAA, branched chain amino acids; BMI, body mass index; HDL, high density lipoprotein; HOMA-IR, homeostatic model assessment of insulin resistance; LDL, low density lipoprotein; LP-IR, Lipoprotein Insulin Resistance Index.*

**FIGURE 1:**
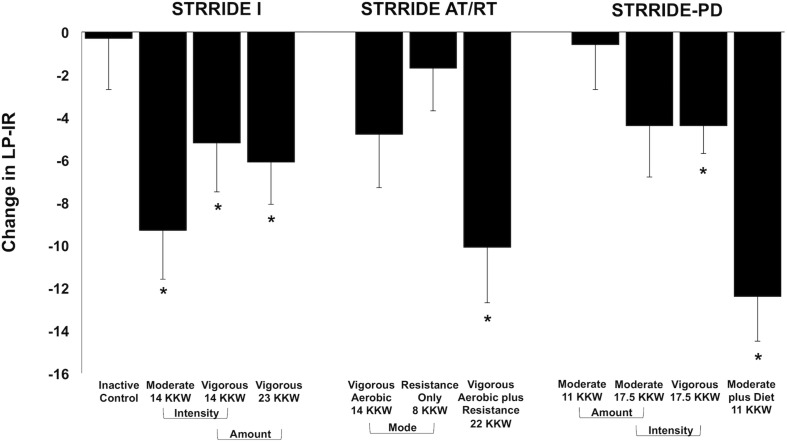
Change in Lipoprotein Insulin Resistance Index (LP-IR) following intervention across all groups from STRRIDE I, STRRIDE AT/RT, and STRRIDE-PD. Moderate and vigorous refer to prescribed aerobic exercise intensity. Prescribed amount of exercise represented by kcal/kg of body weight/week (KKW). *Denotes significant within-group change following intervention.

Figure 1 displays change scores across all groups for LP-IR. The inactive control group did not significantly change LP-IR. After training, six of the 10 exercise groups significantly improved LP-IR, ranging from −4.4 ± 8.2 to −12.4 ± 14.1 points. In STRRIDE I, all three aerobic training programs resulted in significant decreases in LP-IR. The low amount/moderate intensity aerobic exercise group had the greatest improvement in LP-IR (−9.3 ± 15.5 points; *p* = 0.0003), which was significantly different from the inactive control group (−0.3 ± 17.4 points; *p* = 0.9) (*p* = 0.04 for difference among groups). In STRRIDE AT/RT, the aerobic plus resistance training program resulted in a robust LP-IR improvement (−10.1 ± 16.8 points; *p* = 0.0003), which was significantly different from the resistance training only group (−1.7 ± 14.0 points; *p* = 0.39) (*p* = 0.04 for difference among groups). In STRRIDE-PD, the clinical lifestyle program resulted in the most robust LP-IR change (−12.4 ± 14.1 points; *p* < 0.0001), and this change was significantly greater than all other groups (*p* = 0.0009 for difference among groups). Both of the high amount aerobic exercise groups had similar magnitudes of change; however, only the high amount/vigorous intensity group reached statistical significance [high amount/vigorous intensity: −4.4 ± 8.2 points (*p* = 0.0016); high amount/moderate intensity: −4.4 ± 16.2 points (*p* = 0.07)].

Across all three STRRIDE cohorts, the only group to experience a significant change in total branched chain amino acid concentration was the clinical lifestyle group from STRRIDE-PD (−16.2 ± 50.9 μmol/L; *p* = 0.043). When assessing the concentrations of valine and leucine individually, no group experienced a significant change (Table 2).

**TABLE 2:** Baseline and change from baseline in LP-IR, BCAA, and Diabetes Risk Index within the various intervention groups in (A) STRRIDE I, (B) STRRIDE AT/RT, and (C) STRRIDE-PD.

A	Inactive control			Low amount/moderate			Low amount/vigorous			High amount/vigorous		
	(*n* = 53)			intensity (*n* = 44)			intensity (*n* = 48)			intensity (*n* = 49)		
				
	Baseline	Change	*p* value	Baseline	Change	*p* value	Baseline	Change	*p* value	Baseline	Change	*p* value
LP-IR	53.1 ± 25.2	−0.3 ± 17.4	0.90	60.0 ± 25.0	−**9.3 ± 15.5**	**0.0003**	52.1 ± 25.4	−**5.2 ± 15.9**	**0.03**	54.7 ± 24.3	−**6.1 ± 13.8**	**0.003**
Total BCAA	380.4 ± 102.2	2.2 ± 67.7	0.81	393.4 ± 99.8	−4.6 ± 76.5	0.69	405.0 ± 87.0	0.9 ± 65.9	0.93	407.8 ± 104.8	10.7 ± 68.6	0.28
Valine	200.0 ± 49.4	3.6 ± 38.7	0.50	213.8 ± 48.3	−2.5 ± 43.5	0.71	212.3 ± 44.6	3.2 ± 37.4	0.56	215.4 ± 53.8	4.1 ± 44.6	0.52
Leucine	141.7 ± 46.7	−0.6 ± 26.3	0.86	141.3 ± 43.4	−1.6 ± 29.0	0.72	152.0 ± 39.8	−1.6 ± 25.6	0.67	153.1 ± 44.3	3.2 ± 22.3	0.32
DRI	39.9 ± 21.3	−0.2 ± 12.0	0.92	43.7 ± 18.3	−**4.7 ± 14.4**	**0.036**	43.0 ± 20.0	−2.6 ± 8.9	0.052	44.2 ± 20.4	−1.5 ± 12.2	0.41

**B**	**RT only (*n* = 50)**			**AT only (*n* = 46)**			**AT/RT (*n* = 43)**					
			
	**Baseline**	**Change**	***p* value**	**Baseline**	**Change**	***p* value**	**Baseline**	**Change**	***p* value**			

LP-IR	48.7 ± 24.4	−1.7 ± 14.0	0.39	54.0 ± 25.5	−4.8 ± 17.2	0.07	54.3 ± 20.4	−**10.1 ± 16.8**	**0.0003**			
Total BCAA	394.0 ± 84.0	−3.7 ± 66.3	0.70	410.6 ± 77.5	0.6 ± 57.8	0.94	412.5 ± 84.7	−13.3 ± 69.7	0.22			
Valine	223.7 ± 36.3	0.9 ± 33.4	0.85	230.7 ± 36.4	4.0 ± 36.4	0.46	229.5 ± 36.6	−3.6 ± 36.3	0.52			
Leucine	115.5 ± 36.4	−1.7 ± 28.9	0.68	124.0 ± 33.0	−3.7 ± 26.6	0.35	124.5 ± 31.7	−3.5 ± 26.4	0.39			
DRI	40.8 ± 18.2	−0.8 ± 12.2	0.63	45.9 ± 19.2	−2.9 ± 11.9	0.10	46.0 ± 16.3	−**6.2 ± 11.0**	**0.0007**			

**C**	**Low amount/moderate**			**High amount/moderate**			**High amount/vigorous**			**Clinical lifestyle**		
	**intensity (*n* = 42)**			**intensity (*n* = 45)**			**intensity (*n* = 40)**			**Clinical lifestyle (*n* = 43)**		
				
	**Baseline**	**Change**	***p* value**	**Baseline**	**Change**	***p* value**	**Baseline**	**Change**	***p* value**	**Baseline**	**Change**	***p* value**

LP-IR	54.5 ± 22.4	−0.6 ± 13.9	0.78	55.2 ± 22.6	−4.4 ± 16.2	0.07	55.2 ± 22.4	−**4.4 ± 8.2**	**0.0016**	57.1 ± 18.3	−**12.4 ± 14.1**	**<0.0001**
Total BCAA	379.0 ± 63.5	−2.8 ± 52.2	0.73	383.0 ± 73.7	2.9 ± 46.6	0.68	386.7 ± 71.1	−6.0 ± 46.2	0.42	387.2 ± 83.9	−**16.2 ± 50.9**	**0.043**
Valine	216.3 ± 33.8	−3.5 ± 32.0	0.49	221.4 ± 36.7	2.2 ± 29.4	0.62	223.1 ± 39.6	−1.6 ± 28.5	0.73	219.9 ± 41.8	−6.3 ± 31.8	0.20
Leucine	117.4 ± 21.8	1.3 ± 21.2	0.70	117.5 ± 29.2	0.9 ± 22.6	0.78	117.7 ± 23.5	−0.8 ± 19.8	0.81	118.8 ± 30.1	−6.1 ± 21.5	0.07
DRI	37.9 ± 16.8	−0.6 ± 10.0	0.71	38.5 ± 18.3	−1.4 ± 11.5	0.43	39.1 ± 17.1	−**2.8 ± 8.2**	**0.039**	39.2 ± 16.8	−**8.3 ± 10.4**	**<0.0001**

*Results with *p* values < 0.05 are in bold. AT, aerobic training; AT only exercise prescription equivalent to Low Amount/Vigorous Intensity group from STRRIDE I; AT/RT, aerobic plus resistance training group; BCAA, branched chain amino acids (μmol/L); DRI, Diabetes Risk Index; LP-IR, Lipoprotein Insulin Resistance Index; PD, prediabetes; RT, resistance training.*

**FIGURE 2:**
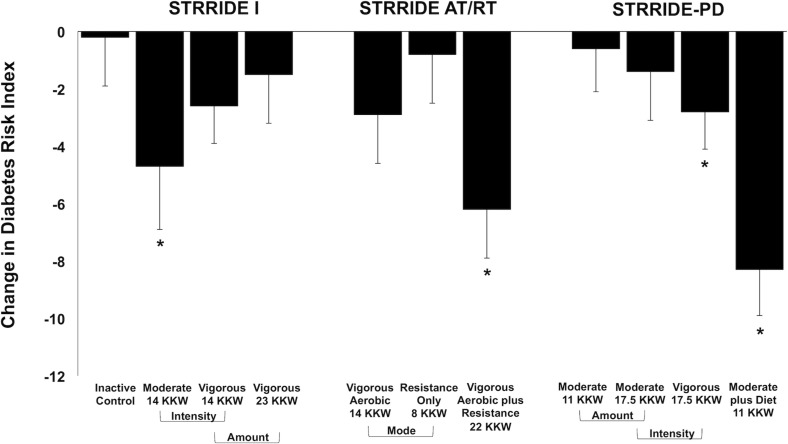
Change in Diabetes Risk Index following intervention across all groups from STRRIDE I, STRRIDE AT/RT, and STRRIDE-PD. Moderate and vigorous refer to prescribed aerobic exercise intensity. Prescribed amount of exercise represented by kcal/kg of body weight/week (KKW). *Denotes significant within-group change following intervention.

Change scores across all groups for the Diabetes Risk Index are shown in Figure 2. The inactive control group did not significantly change the Diabetes Risk Index. After training, four of the 10 exercise groups significantly improved the Diabetes Risk Index, ranging from −2.8 ± 8.2 to −8.3 ± 10.4 points. In STRRIDE I, both low amount/moderate intensity and low amount/vigorous intensity groups improved the Diabetes Risk Index (−4.7 ± 14.4 points, *p* = 0.036; −2.6 ± 8.9 points, *p* = 0.052, respectively); however, these changes were only statistically significant for the low amount/moderate intensity group. In STRRIDE AT/RT, only the aerobic plus resistance training group experienced a significant beneficial change in the Diabetes Risk Index (−6.2 ± 11.0 points; *p* = 0.0007). Finally, in STRRIDE-PD, both the high amount/vigorous intensity group (−2.8 ± 8.2 points; *p* = 0.039) and the clinical lifestyle group (−8.3 ± 10.4 points; *p* < 0.0001) experienced significant decreases in the Diabetes Risk Index. The change in the clinical lifestyle group was significantly greater than the change in both the moderate intensity groups [low amount/moderate intensity: −0.6 ± 10.0 points (*p* = 0.71); high amount/moderate intensity: −1.4 ± 11.5 points (*p* = 0.43)].

## Discussion

The STRRIDE trials provided a unique opportunity to assess the effects of 10 distinct exercise interventions on LP-IR and the Diabetes Risk Index, two nuclear magnetic resonance-derived multimarkers of insulin resistance and type 2 diabetes risk. Across all three studies, a majority of the STRRIDE interventions elicited notable beneficial changes in LP-IR, as six interventions resulted in significant improvements. Additionally, four interventions significantly improved Diabetes Risk Index scores. As both LP-IR and the Diabetes Risk Index were sensitive to change following exercise training, our findings highlight the clinical utility of these novel multimarkers to assess alterations in disease risk with lifestyle intervention.

Our findings from STRRIDE I and STRRIDE-PD suggest differential aerobic exercise training effects based upon glycemic status. For STRRIDE I, which consisted of participants with dyslipidemia and normal blood glucose concentrations, the low amount/moderate intensity intervention was the most beneficial training program for both LP-IR and the Diabetes Risk Index. Conversely, the effect of the low amount/moderate intensity intervention in STRRIDE-PD was negligible for both outcomes. For participants with prediabetes, a greater amount of aerobic exercise was needed to elicit improvements in LP-IR and the Diabetes Risk Index in the absence of dietary intervention. Thus, for those with a glycemic status closer to type 2 diabetes, changing insulin resistance and type 2 diabetes risk may be more challenging with aerobic exercise alone.

The clinical lifestyle group in STRRIDE-PD mimicked the Diabetes Prevention Program, allowing us to investigate how much of the “gold standard” effect is attained with different amounts, intensities, and modes of exercise. STRRIDE-PD’s most effective exercise-only intervention for LP-IR and the Diabetes Risk Index – high amount/vigorous intensity aerobic training – achieved only 35.5 and 33.7% of the clinical lifestyle results. Notably, greater magnitudes of the clinical lifestyle effect were observed in STRRIDE I and STRRIDE AT/RT. STRRIDE I’s low amount/moderate intensity aerobic intervention produced 75.0 and 56.6% of the clinical lifestyle effect for LP-IR and the Diabetes Risk Index. In STRRIDE AT/RT, the aerobic plus resistance training intervention resulted in 81.5 and 74.7% of the clinical lifestyle effect for LP-IR and the Diabetes Risk Index. Thus, achieving a large proportion of the “gold standard” effect with exercise alone may require intervention earlier in the progression to type 2 diabetes.

Across the STRRIDE trials, the training effects observed for LP-IR reflect previously published findings on traditional markers of insulin action (e.g., fasting insulin, HOMA-IR, insulin sensitivity index from IVGTT, and Matsuda index from OGTT) (Houmard et al., 2004; AbouAssi et al., 2015; Slentz et al., 2016). As expected, baseline LP-IR was moderately correlated with baseline HOMA-IR, insulin sensitivity index, the reciprocal of insulin sensitivity index (i.e., 1/insulin sensitivity index as a proxy of insulin resistance), and Matsuda index (Supplementary Table 1). Consistent with previously reported correlations of LP-IR with HOMA-IR and glucose disposal rate (Shalaurova et al., 2014), the strength of these associations illustrate the various mechanisms of overall insulin resistance each measurement assesses. In STRRIDE I, all three of the aerobic training interventions elicited beneficial reductions in LP-IR, while LP-IR in the inactive control group did not change (Figure 1). Similarly, all three aerobic interventions significantly reduced fasting insulin and HOMA-IR, and improved insulin sensitivity index. On the other hand, the inactive control group experienced significant worsening of fasting insulin and HOMA-IR, and no change in insulin sensitivity index (Houmard et al., 2004). IVGTT-derived measures of insulin sensitivity are considered to reflect skeletal muscle or peripheral insulin sensitivity, while HOMA-IR is thought to reflect hepatic insulin sensitivity (DeFronzo and Tripathy, 2009). The pattern of response of LP-IR reflects that of IVGTT-derived insulin sensitivity in STRRIDE I; however, change in LP-IR was not correlated with change in insulin sensitivity index (ρ = −0.12; *p* = 0.10; Supplementary Table 1). Thus, the lipoprotein-based marker LP-IR most likely reflects another mechanism of insulin resistance – adipose tissue insulin resistance – where the presence of high insulin levels impairs the suppression of lipolysis (Gastaldelli et al., 2017). Subsequently, the elevated levels of free fatty acids can impair muscle signaling, promote hepatic gluconeogenesis, and impair glucose-stimulated insulin response, which impact the transition from normal glucose tolerance to type 2 diabetes (Ferrannini et al., 1983; Roden et al., 1996, 2000; Kashyap et al., 2003; Belfort et al., 2005; Allister et al., 2015). The same pattern of response was observed when assessing our findings from STRRIDE AT/RT. We found the combination of aerobic plus resistance training resulted in a notable synergistic effect on LP-IR, which mimicked the robust, synergistic effect previously documented for the IVGTT-derived insulin sensitivity index (AbouAssi et al., 2015). Similar to STRRIDE I, there was a weak correlation between change in LP-IR with change in insulin sensitivity index (ρ = −0.22; *p* = 0.02; Supplementary Table 1). This is an important finding and confirms the concept of preferential tissue-specificity of the LP-IR effect. Finally, in STRRIDE-PD, incorporating dietary intervention with aerobic training resulted in the most robust improvement in LP-IR; without dietary intervention, a greater amount of aerobic exercise was required to elicit a beneficial LP-IR change in those with prediabetes. We observed similar patterns for fasting insulin and HOMA-IR. However, LP-IR findings did not mirror the patterns of response for OGTT-derived markers, as all four intervention groups experienced significant improvements in insulin area under the curve and Matsuda index (Slentz et al., 2016). Further analysis showed that change in LP-IR was weakly correlated with change in Matsuda index (ρ = −0.32; *p* < 0.0001; Supplementary Table 1). Likely, these differences in measures reflect that OGTTs assess more complex, integrated physiology capturing hepatic and muscle insulin sensitivities, as well as influences of gut hormones and neurotransmitters (Ahren et al., 2008). Thus, improvements in any of these components will be reflected in beneficial OGTT changes, while other measures, including LP-IR, are more specific. In summary, LP-IR adaptations likely reflect improvements in the adipose tissue-related mechanism of insulin action, which contributes to both skeletal muscle and hepatic mechanisms to improve whole-body insulin sensitivity.

As only four STRRIDE interventions resulted in significant Diabetes Risk Index improvements (Figure 2), the changes in the scores were not as consistent as those observed for LP-IR. Among the groups experiencing significant reductions in the Diabetes Risk Index, the response appeared to be driven largely by decreases in insulin resistance (LP-IR), but not by dysmetabolism (branched chain amino acids). Only the clinical lifestyle group, which incorporated dietary intervention and weight loss, exhibited reductions in both Diabetes Risk Index components (LP-IR and branched chain amino acids). Although we expected weight loss to be an important contributor to changes in plasma branched chain amino acid concentrations (Felig et al., 1969), we did not expect null findings across all of the exercise-only intervention groups. The greatest weight loss in any of the exercise-only groups was 2.2% on average (data not shown). The clinical lifestyle group exhibited an average 7.1% reduction in body weight; however, their reduction in branched chain amino acid concentration was just beyond the statistical significance level. Thus, weight reduction greater than 7.0% may be required in lifestyle interventions that include exercise, diet, or combination thereof in order to elicit a significant reduction in branched chain amino acids.

As demonstrated by the standard deviations in Table 2, the mean changes in LP-IR and the Diabetes Risk Index corresponded to a relatively high variability of response across groups. Although heterogeneity is expected and characteristic of a Gaussian distribution, we created waterfall plots to assess the distribution of individual responses of LP-IR and the Diabetes Risk Index. In STRRIDE AT/RT, the aerobic plus resistance training group significantly and robustly improved LP-IR at the group level, while the aerobic and resistance training only groups did not significantly change from baseline. However, when plotting individual change scores ordered by magnitude of change, all three groups display a broad range of responses, from positive to negative (Supplementary Figure 1); even the group with the greatest average improvement included participants who either had no significant improvement or a worsening of LP-IR (Supplementary Figure 1C). The same pattern is observed when assessing the individual change scores for the Diabetes Risk Index across the four groups in STRRIDE-PD; even the clinical lifestyle group with the robust average improvement included participants with either no change or a worsening of score (Supplementary Figure 2D). Despite an overall broad range of responsiveness, the waterfall plots illustrate that – as compared to the other groups in their respective cohorts – a larger proportion of participants in the aerobic plus resistance and clinical lifestyle groups fell within the distribution of beneficial responses for LP-IR and the Diabetes Risk Index.

Previous studies have demonstrated the comparability of LP-IR to traditional markers for determining a patient’s insulin resistance state (Mackey et al., 2015; Dugani et al., 2016; Harada et al., 2017; Flores-Guerrero et al., 2019). However, only one study has assessed the effects of comprehensive lifestyle programs incorporating exercise on LP-IR. Ellsworth et al. (2016) evaluated two clinical lifestyle programs – differing in dietary stringency, exercise intensity, and time commitment – in patients with type 2 diabetes, coronary artery disease, or significant cardiometabolic risk factors. At the end of the year-long programs, the “intensive” and “moderate” interventions led to 13.3 and 8.8% reductions in LP-IR, respectively; for comparison, our STRRIDE interventions resulted in significant LP-IR reductions between 8.0 and 21.8%. Interestingly, Ellsworth et al. (2016) did not find a significant correlation between change in exercise minutes per week during the intervention and change in LP-IR (*r* = −0.112; *p* = 0.138). However, both clinical lifestyle programs relied on self-reported data and did not employ a structured exercise program. Therefore, we are the first to investigate the effects of supervised exercise interventions on both LP-IR and the Diabetes Risk Index.

### Strengths and Limitations

Hypertriglyceridemia resulting from reasons other than insulin resistance (e.g., familial combined hyperlipidemia) may possibly cause elevated LP-IR and/or the Diabetes Risk Index scores in the absence of clinical insulin resistance. However, as the average baseline fasting triglyceride levels were modest, ranging from 128.8 ± 70.8 mg/dL (STRRIDE-PD) to 158.6 ± 98.1 mg/dL (STRRIDE I) across the three cohorts, many participants in STRRIDE presenting with this clinical scenario as a cause of elevated LP-IR or the Diabetes Risk Index is unlikely. For change in LP-IR, we were unable to detect the distinct amount and intensity effects originally documented for STRRIDE I’s individual lipid and lipoprotein outcomes, possibly because LP-IR is a multimarker composed of six lipoprotein parameters that respond differently to exercise amount and intensity. For example, greater amounts of aerobic exercise drive improvements in HDL particles, while moderate intensity aerobic exercise is more beneficial for VLDL particles compared to vigorous intensity exercise (Kraus et al., 2002). Although the individual effects of exercise amount and intensity may not be discernible in the multi-component biomarker of LP-IR, the change in the composite score clearly reflects the net result of several exercise intervention effects, demonstrated by the fact that all aerobic interventions in STRRIDE I significantly improved LP-IR.

Aerobic exercise prescriptions were calculated at 50 and 75% of V̇O_2peak_ (STRRIDE I and STRRIDE AT/RT) or V̇O_2reserve_ (STRRIDE-PD) for moderate and vigorous intensity exercise groups – the middle upper percentages of the prescribed intensity ranges. For intervention feasibility, participants exercised within a target heart rate range that corresponded to their intensity prescription (Kraus et al., 2001). We recognize the inherent individual variability of V̇O_2_ kinetics; thus, normalizing exercise intensity based on V̇O_2peak_ may not have resulted in homogenous moderate or vigorous intensity exercise classifications for all participants within their respective groups (Iannetta et al., 2020).

LP-IR and the Diabetes Risk Index were developed to address the clinical need for simple, cost-effective diagnostic tools for early identification of patients at greater risk for type 2 diabetes development. As LP-IR and the Diabetes Risk Index were sensitive to change following exercise intervention, our study demonstrates the added clinical value of utilizing these new multimarkers to monitor disease progression.

We also recognize that LP-IR and the Diabetes Risk Index are primarily markers of type 2 diabetes risk. As the STRRIDE studies were not diabetes outcomes studies, we can only infer the effects of the interventions on true type 2 diabetes incidence and progression. However, our group is currently conducting a series of long-term follow-up studies on the STRRIDE cohorts (i.e., the STRRIDE Reunion studies), which include medical histories and fasted plasma samples approximately 10 years after STRRIDE participation. These Reunion studies will allow us to ascertain type 2 diabetes prevalence, examine whether baseline LP-IR and the Diabetes Risk Index are related to future type 2 diabetes development, and investigate legacy effects of the different interventions on 10-year LP-IR and Diabetes Risk Index scores.

## Conclusion

Overall, our results show the power of multiple exercise interventions – varying in amount, intensity, and mode – to improve both LP-IR and Diabetes Risk Index scores, with LP-IR exhibiting greater sensitivity to change. Adding resistance to aerobic training elicited the greatest beneficial effect for individuals with dyslipidemia and normal glycemic status. For those with prediabetes, incorporating dietary intervention with aerobic training resulted in the most robust improvements in LP-IR and the Diabetes Risk Index. Ultimately, our results not only provide evidence for the clinical utility of these multimarkers to assess state of insulin resistance and type 2 diabetes risk, but also demonstrate the profound impact different exercise interventions can have on both traditional and novel markers of cardiometabolic disease risk.

## Data Availability Statement

The raw data supporting the conclusions of this article will be made available by the authors, without undue reservation. Requests to access these datasets should be directed to WK.

## Ethics Statement

The studies involving human participants were reviewed and approved by Duke University and East Carolina University Institutional Review Boards. The patients/participants provided their written informed consent to participate in this study.

## Author Contributions

LR and WK developed the aims and hypothesis of the study. LR performed statistical analysis and drafted the manuscript. All authors contributed to study concept and design, acquisition or interpretation of the data, and drafting or critical revision of the manuscript for important intellectual content. All authors approved the final version of the manuscript.

## Conflict of Interest

IS, JO, and MC are employees of LabCorp. The remaining authors declare that the research was conducted in the absence of any commercial or financial relationships that could be construed as a potential conflict of interest.

## Supplementary Material

Supplementary Figure 1 Distribution of individual responses for change in Lipoprotein Insulin Resistance Index (LP-IR) following 8 months of exercise training in STRRIDE AT/RT. Each bar represents an individual participant’s change score. **(A)** Aerobic training only, **(B)** resistance training only, and **(C)** aerobic plus resistance training.

Supplementary Figure 2 Distribution of individual responses for change in Diabetes Risk Index following 6 months of exercise training in STRRIDE-PD. Each bar represents an individual participant’s change score. **(A)** Low amount/moderate intensity aerobic training, **(B)** high amount/moderate intensity aerobic training, **(C)** high amount/vigorous intensity aerobic training, and **(D)** low amount/moderate intensity aerobic training plus diet.

## References

[B1] AbouAssi H. Slentz C. A. Mikus C. R. Tanner C. J. Bateman L. A. Willis L. H. (2015). The effects of aerobic, resistance, and combination training on insulin sensitivity and secretion in overweight adults from STRRIDE AT/RT: a randomized trial. *J. Appl. Physiol.* 118 1474–1482. 10.1152/japplphysiol.00509.2014 25882384 PMC4469920

[B2] Ahren B. Winzell M. S. Pacini G. (2008). The augmenting effect on insulin secretion by oral versus intravenous glucose is exaggerated by high-fat diet in mice. *J. Endocrinol.* 197 181–187. 10.1677/JOE-07-0460 18372244

[B3] Allister C. A. Liu L. F. Lamendola C. A. Craig C. M. Cushman S. W. Hellerstein M. K. (2015). In vivo 2H2O administration reveals impaired triglyceride storage in adipose tissue of insulin-resistant humans. *J. Lipid Res.* 56 435–439. 10.1194/jlr.M052860 25418322 PMC4306696

[B4] Belfort R. Mandarino L. Kashyap S. Wirfel K. Pratipanawatr T. Berria R. (2005). Dose-response effect of elevated plasma free fatty acid on insulin signaling. *Diabetes* 54 1640–1648. 10.2337/diabetes.54.6.1640 15919784

[B5] Berends A. M. A. Buitenwerf E. Gruppen E. G. Sluiter W. J. Bakker S. J. L. Connelly M. A. (2019). Primary aldosteronism is associated with decreased low-density and high-density lipoprotein particle concentrations and increased GlycA, a pro-inflammatory glycoprotein biomarker. *Clin. Endocrinol. (Oxf.)* 90 79–87. 10.1111/cen.13891 30372543

[B6] Defronzo R. A. (2009). Banting lecture. from the triumvirate to the ominous octet: a new paradigm for the treatment of type 2 diabetes mellitus. *Diabetes* 58 773–795. 10.2337/db09-9028 19336687 PMC2661582

[B7] DeFronzo R. A. Tripathy D. (2009). Skeletal muscle insulin resistance is the primary defect in type 2 diabetes. *Diabetes Care* 32(Suppl. 2) S157–S163. 10.2337/dc09-S302 19875544 PMC2811436

[B8] Dugani S. B. Akinkuolie A. O. Paynter N. Glynn R. J. Ridker P. M. Mora S. (2016). Association of lipoproteins. insulin resistance, and rosuvastatin with incident type 2 diabetes mellitus : secondary analysis of a randomized clinical trial. *JAMA Cardiol.* 1 136–145. 10.1001/jamacardio.2016.0096 27347563 PMC4918085

[B9] Duscha B. D. Slentz C. A. Johnson J. L. Houmard J. A. Bensimhon D. R. Knetzger K. J. (2005). Effects of exercise training amount and intensity on peak oxygen consumption in middle-age men and women at risk for cardiovascular disease. *Chest* 128 2788–2793. 10.1378/chest.128.4.2788 16236956

[B10] Ellsworth D. L. Costantino N. S. Blackburn H. L. Engler R. J. Kashani M. Vernalis M. N. (2016). Lifestyle modification interventions differing in intensity and dietary stringency improve insulin resistance through changes in lipoprotein profiles. *Obes. Sci. Pract.* 2 282–292. 10.1002/osp4.54 27708845 PMC5043634

[B11] Felig P. Marliss E. Cahill G. F. Jr. (1969). Plasma amino acid levels and insulin secretion in obesity. *N. Engl. J. Med.* 281 811–816. 10.1056/NEJM196910092811503 5809519

[B12] Ferrannini E. Barrett E. J. Bevilacqua S. DeFronzo R. A. (1983). Effect of fatty acids on glucose production and utilization in man. *J. Clin. Invest.* 72 1737–1747. 10.1172/JCI111133 6138367 PMC370462

[B13] Flores-Guerrero J. L. Connelly M. A. Shalaurova I. Gruppen E. G. Kieneker L. M. Dullaart R. P. F. (2019). Lipoprotein insulin resistance index, a high-throughput measure of insulin resistance, is associated with incident type II diabetes mellitus in the prevention of renal and vascular end-stage disease study. *J. Clin. Lipidol.* 129–137.e1. 10.1016/j.jacl.2018.11.009 30591414

[B14] Flores-Guerrero J. L. Gruppen E. G. Connelly M. A. Shalaurova I. Otvos J. D. Garcia E. (2020). A newly developed diabetes risk index, based on lipoprotein subfractions and branched chain amino acids, is associated with incident type 2 diabetes mellitus in the PREVEND cohort. *J. Clin. Med.* 9:2781 10.3390/jcm9092781 PMC7563197 32867285

[B15] Flores-Guerrero J. L. Oste M. C. J. Kieneker L. M. Gruppen E. G. Wolak-Dinsmore J. Otvos J. D. (2018). Plasma branched-chain amino acids and risk of incident type 2 diabetes: results from the PREVEND prospective cohort study. *J. Clin. Med.* 7:513. 10.3390/jcm7120513 30518023 PMC6306832

[B16] Frazier-Wood A. C. Garvey W. T. Dall T. Honigberg R. Pourfarzib R. (2012). Opportunities for using lipoprotein subclass profile by nuclear magnetic resonance spectroscopy in assessing insulin resistance and diabetes prediction. *Metab. Syndr. Relat. Disord.* 10 244–251. 10.1089/met.2011.0148 22533466 PMC3409454

[B17] Gastaldelli A. Gaggini M. DeFronzo R. A. (2017). Role of adipose tissue insulin resistance in the natural history of type 2 diabetes: results from the San Antonio metabolism study. *Diabetes* 66 815–822. 10.2337/db16-1167 28052966

[B18] Harada P. H. N. Demler O. V. Dugani S. B. Akinkuolie A. O. Moorthy M. V. Ridker P. M. (2017). Lipoprotein insulin resistance score and risk of incident diabetes during extended follow-up of 20 years: the Women’s health study. *J. Clin. Lipidol.* 11 1257–1267.e2. 10.1016/j.jacl.2017.06.008 28733174 PMC5644504

[B19] Holecek M. (2018). Branched-chain amino acids in health and disease: metabolism, alterations in blood plasma, and as supplements. *Nutr. Metab. (Lond.)* 15:33. 10.1186/s12986-018-0271-1 29755574 PMC5934885

[B20] Houmard J. Tanner C. Slentz C. Duscha B. McCartney J. Kraus W. (2004). Effect of the volume and intensity of exercise training on insulin sensitivity. *J. Appl. Physiol.* 96 101–106. 12972442 10.1152/japplphysiol.00707.2003

[B21] Iannetta D. Inglis E. C. Mattu A. T. Fontana F. Y. Pogliaghi S. Keir D. A. (2020). A critical evaluation of current methods for exercise prescription in women and men. *Med. Sci. Sports Exer.* 52 466–473. 10.1249/mss.0000000000002147 31479001

[B22] Jeyarajah E. J. Cromwell W. C. Otvos J. D. (2006). Lipoprotein particle analysis by nuclear magnetic resonance spectroscopy. *Clin. Lab. Med.* 26 847–870. 10.1016/j.cll.2006.07.006 17110242

[B23] Kashyap S. Belfort R. Gastaldelli A. Pratipanawatr T. Berria R. Pratipanawatr W. (2003). A sustained increase in plasma free fatty acids impairs insulin secretion in nondiabetic subjects genetically predisposed to develop type 2 diabetes. *Diabetes* 52 2461–2474. 10.2337/diabetes.52.10.2461 14514628

[B24] Kinzer A. B. Shamburek R. D. Lightbourne M. Muniyappa R. Brown R. J. (2019). Advanced lipoprotein analysis shows atherogenic lipid profile that improves after metreleptin in patients with lipodystrophy. *J. Endocr. Soc.* 3 1503–1517. 10.1210/js.2019-00103 31620670 PMC6676079

[B25] Knowler W. C. Barrett-Connor E. Fowler S. E. Hamman R. F. Lachin J. M. Walker E. A. (2002). Reduction in the incidence of type 2 diabetes with lifestyle intervention or metformin. *N. Engl. J. Med.* 346 393–403. 10.1056/NEJMoa012512 11832527 PMC1370926

[B26] Kraus W. E. Houmard J. A. Duscha B. D. Knetzger K. J. Wharton M. B. McCartney J. S. (2002). Effects of the amount and intensity of exercise on plasma lipoproteins. *N. Engl. J. Med.* 347 1483–1492. 10.1056/NEJMoa020194 12421890

[B27] Kraus W. E. Torgan C. E. Duscha B. D. Norris J. Brown S. A. Cobb F. R. (2001). Studies of a targeted risk reduction intervention through defined exercise (STRRIDE). *Med. Sci. Sports Exerc.* 33 1774–1784. 11581566 10.1097/00005768-200110000-00025

[B28] Lynch C. J. Adams S. H. (2014). Branched-chain amino acids in metabolic signalling and insulin resistance. *Nat. Rev. Endocrinol.* 10 723–736. 10.1038/nrendo.2014.171 25287287 PMC4424797

[B29] Mackey R. H. Mora S. Bertoni A. G. Wassel C. L. Carnethon M. R. Sibley C. T. (2015). Lipoprotein particles and incident type 2 diabetes in the multi-ethnic study of atherosclerosis. *Diabetes Care* 38 628–636. 10.2337/dc14-0645 25592196 PMC4370328

[B30] Makri A. Cheung A. Sinaii N. Remaley A. T. Sampson M. Keil M. (2019). Lipoprotein particles in patients with pediatric Cushing disease and possible cardiovascular risks. *Pediatr. Res.* 86 375–381. 10.1038/s41390-019-0438-0 31112990 PMC6702083

[B31] Matsuda M. DeFronzo R. A. (1999). Insulin sensitivity indices obtained from oral glucose tolerance testing: comparison with the euglycemic insulin clamp. *Diabetes Care* 22 1462–1470. 10.2337/diacare.22.9.1462 10480510

[B32] Newgard C. B. (2012). Interplay between lipids and branched-chain amino acids in development of insulin resistance. *Cell Metab.* 15 606–614. 10.1016/j.cmet.2012.01.024 22560213 PMC3695706

[B33] Newgard C. B. An J. Bain J. R. Muehlbauer M. J. Stevens R. D. Lien L. F. (2009). A branched-chain amino acid-related metabolic signature that differentiates obese and lean humans and contributes to insulin resistance. *Cell Metab.* 9 311–326. 10.1016/j.cmet.2009.02.002 19356713 PMC3640280

[B34] Roden M. Price T. B. Perseghin G. Petersen K. F. Rothman D. L. Cline G. W. (1996). Mechanism of free fatty acid-induced insulin resistance in humans. *J. Clin. Invest.* 97 2859–2865. 10.1172/JCI118742 8675698 PMC507380

[B35] Roden M. Stingl H. Chandramouli V. Schumann W. C. Hofer A. Landau B. R. (2000). Effects of free fatty acid elevation on postabsorptive endogenous glucose production and gluconeogenesis in humans. *Diabetes* 49 701–707. 10.2337/diabetes.49.5.701 10905476

[B36] Shalaurova I. Connelly M. A. Garvey W. T. Otvos J. D. (2014). Lipoprotein insulin resistance index: a lipoprotein particle-derived measure of insulin resistance. *Metab. Syndr. Relat. Disord.* 12 422–429. 10.1089/met.2014.0050 24959989 PMC4175429

[B37] Slentz C. A. Bateman L. Willis L. Granville E. Piner L. Samsa G. (2016). Effects of exercise training alone vs combined exercise and nutritional lifestyle intervention on glucose homeostasis in prediabetic individuals: a randomized controlled trial. *Diabetologia* 59 2088–2098. 27421729 10.1007/s00125-016-4051-z PMC5026926

[B38] Slentz C. A. Bateman L. A. Willis L. H. Shields A. T. Tanner C. J. Piner L. W. (2011). Effects of aerobic vs. resistance training on visceral and liver fat stores, liver enzymes, and insulin resistance by HOMA in overweight adults from STRRIDE AT/RT. *Am. J. Physiol. Endocrinol. Metab.* 301 E1033–E1039. 10.1152/ajpendo.00291.2011 21846904 PMC3214001

[B39] Wolak-Dinsmore J. Gruppen E. G. Shalaurova I. Matyus S. P. Grant R. P. Gegen R. (2018). A novel NMR-based assay to measure circulating concentrations of branched-chain amino acids: elevation in subjects with type 2 diabetes mellitus and association with carotid intima media thickness. *Clin. Biochem.* 54 92–99. 10.1016/j.clinbiochem.2018.02.001 29432757

[B40] Yoon M. S. (2016). The emerging role of branched-chain amino acids in insulin resistance and metabolism. *Nutrients* 8:405. 10.3390/nu8070405 27376324 PMC4963881

