# Supplementary figures and images for: Effects of Amount, Intensity, and Mode of Exercise Training on Insulin Resistance and Type 2 Diabetes Risk in the STRRIDE Randomized Trials
Source: Front Physiol. 2021 Feb 4;12:626142. doi: 10.3389/fphys.2021.626142 (PMC7892901; doi:10.3389/fphys.2021.626142)

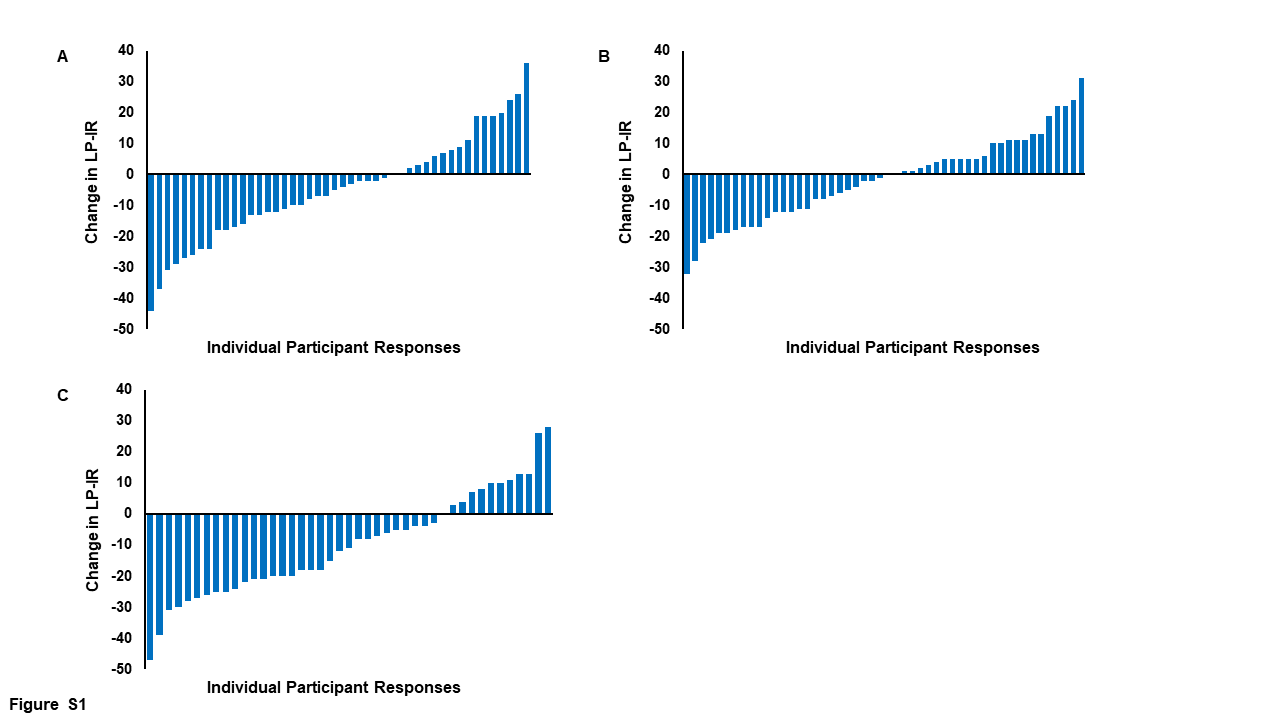

Supplement: Supplementary Figure 1 — Distribution of individual responses for change in Lipoprotein Insulin Resistance Index (LP-IR) following 8 months of exercise training in STRRIDE AT/RT. Each bar represents an individual participant’s change score. (A) Aerobic training only, (B) resistance training only, and (C) aerobic plus resistance training. [file Image_1.TIF]

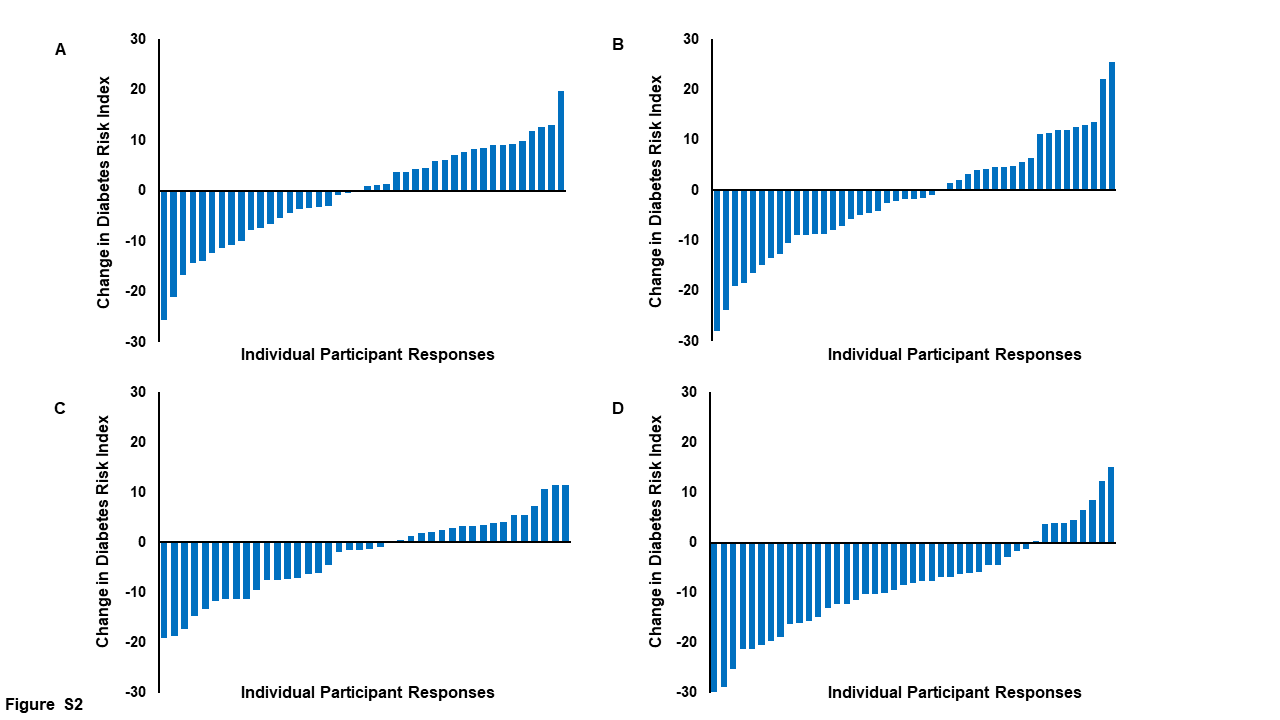

Supplement: Supplementary Figure 2 — Distribution of individual responses for change in Diabetes Risk Index following 6 months of exercise training in STRRIDE-PD. Each bar represents an individual participant’s change score. (A) Low amount/moderate intensity aerobic training, (B) high amount/moderate intensity aerobic training, (C) high amount/vigorous intensity aerobic training, and (D) low amount/moderate intensity aerobic training plus diet. [file Image_2.TIF]
