# Supplementary material for: Effects of Amount, Intensity, and Mode of Exercise Training on Insulin Resistance and Type 2 Diabetes Risk in the STRRIDE Randomized Trials
Source: Front Physiol. 2021 Feb 4;12:626142. doi: 10.3389/fphys.2021.626142 (PMC7892901; doi:10.3389/fphys.2021.626142)
Supplement: Supplementary file 3 [file Table_1.DOCX]

**Table S1.** Spearman correlations of baseline and change from baseline in LP-IR with additional measures of insulin action in overall samples from STRRIDE I, STRRIDE AT/RT, and STRRIDE-PD

|  | **STRRIDE I** | | **STRRIDE AT/RT** | | **STRRIDE-PD** | |
| --- | --- | --- | --- | --- | --- | --- |
|  | ***ρ*** | ***p* value** | ***ρ*** | ***p* value** | ***ρ*** | ***p* value** |
| **Baseline LP-IR and Baseline:** |  |  |  |  |  |  |
| HOMA-IR | 0.411 | *<0.0001* | 0.420 | *<0.0001* | 0.459 | *<0.0001* |
| Insulin sensitivity index | -0.487 | *<0.0001* | -0.462 | *<0.0001* | - | - |
| (Insulin sensitivity index)^-1^ | 0.487 | *<0.0001* | 0.462 | *<0.0001* | - | - |
| Matsuda index | - | - | - | - | -0.486 | *<0.0001* |
| **Change in LP-IR and Change in:** |  |  |  |  |  |  |
| HOMA-IR | 0.159 | *0.028* | 0.358 | *<0.0001* | 0.323 | *<0.0001* |
| Insulin sensitivity index | -0.122 | 0.104 | -0.216 | *0.023* | - | - |
| (Insulin sensitivity index)^-1^ | 0.153 | *0.041* | 0.278 | *0.003* | - | - |
| Matsuda index | - | - | - | - | -0.319 | *<0.0001* |

*Results with p values <0.05 are in italics; HOMA-IR, homeostatic model assessment of insulin resistance; LP-IR, Lipoprotein Insulin Resistance Index; (Insulin sensitivity index)^-1^ represents a proxy of insulin resistance*
